# Medical Notes on Climate, Diseases, Hospitals, and Medical Schools, in France, Italy, and Switzerland; Comprising an Inquiry into the Effects of a Residence in the South of Europe, in Cases of Pulmonary Consumption, and Illustrating the Present State of Medicine in Those Countries

**Published:** 1820-04-01

**Authors:** 


*Medical Notes on Climate, Diseases, Hospitals, and Medical Schools, in France, Italy, and Switzerland; comprising an Inquiry into the Effects of a Residence in the South of Europe, in Cases of Pulmonary Consumption, and illustrating the present State of Medicine in those Countries.* By JAMES CLARK, M. D. London, 1820. pp. 250. Octavo.

WE were lately so completely *taken in* by a goodly volume bearing a title something similar to the one now before us, that it was not without some hesitation that we ventured upon the purchase and perusal of Dr. Clark's. We were indeed, somewhat reassured by the modesty of the title page, and the unpretending humbleness of the preface; but we have waxed too wise and sober on our critical Pharos to risk much of our judgment on such appearances — fronti nulla fides — and it was only after having clandestinely run over, in our bookseller's, some of the pages in the *interior,* that we ventured to order its translation to our own shelves. In this instance, we are happy to say that we have not been disappointed. Dr. Clark gives us more than he promises, but certainly much less than we could wish. Indeed, on many occasions his remarks are so brief and few, that even the title of the volume (Notes) and the apologetical circumstances mentioned in the preface, were insufficient to satisfy our craving for further information, which was only irritated by the little that was afforded us. Upon the whole, however, we have been much pleased and instructed by this little work of Dr. Clark, and we can very conscientiously recommend it to our readers, as containing much useful and interesting matter.

It appears by the Preface (which is in the form of a letter, dated Rome, Nov. 1819, and addressed to Dr. Forbes of Penzance) that the Notes now offered to the public were taken under very varying circumstances (over which the author had frequently no control) of time and convenience, during casual visits to the places noticed; and that they are now presented to the public chiefly at the instance of the author's friends, from a conviction that they contain matter both valuable and interesting, and from apprehension lest events may prevent the accomplishment of the hope he entertains of being hereafter enabled to fill up the outline he has here sketched.

Dr. Clark's book is divided into two parts: the first comprehending his observations on the climate and endemic diseases of some parts of the South of France, Italy, and Switzerland; the second, his account of the state of medicine in some of the hospitals and medical schools of the same countries. In collecting the materials for the first part of his work, the object of Dr. Clark has evidently been to endeavour to ascertain the effect of the climates of the South of France and Italy on those cases of consumption sent thither annually, in such numbers from England; to determine whether this practice is at all useful or injurious; in what cases and stages of the disease it is so; and which, of all the various places resorted to, is the most eligible. In the fulfilment of this plan, in addition to the examination of the topography and local habitudes of the different places, he has naturally been led to inquire into the nature and degree of prevalence of the diseases met with in them, especially of such as have any relation to consumption, or to those qualities of climate found to influence its developement or progress. In this inquiry we are frequently presented with very interesting notices of diseases not particulary connected with the main subject of investigation; also with curious dietetic notices; and valuable practical remarks. We are, also, presented with numerous highly useful directions for the guidance of the invalids in the different places frequented by them, and many cautions and warnings, highly important to this class of sufferers; and which, by the earnestness with which they are endeavoured to be impressed, very advantageously illustrate the feeling and kind-heartedness of the excellent author. In reference, particularly to this part of the book, we consider it as constituting a most excellent guide for the invalid who visits these countries, and earnestly recommend it to all such, and to professional men who may accompany them, as fraught with much that it imports them to know, and which they cannot otherwise acquire than by much and hazardous experiment.

Dr. Clark's experience has led him to form a very low estimate of the beneficial influence of the climate of the South of France and Italy in Pulmonary Consumption. Indeed, the impression left on our minds by an attentive perusal of his work, of the inutility, nay, of the impropriety of sending consumptive patients thither from this country, is very strong, much stronger than that which seems to have been left on the mind of the author himself; certainly infinitely stronger than our previous imperfect information on this matter had led us to entertain. The author sums up his opinion on this subject in the following words, in which we are, in the main, disposed to agree with him.

"To sum up in a few words the opinion I have formed from all the observations I have been enabled to make on the effects of climate in pulmonary consumption, it appears to me, then, that the change of our English climate for a residence in the milder ones of the south of Europe, is much more beneficial as a preventive of the disease, than, I fear, it will ever be found as a means of cure of it when formed. In the young and growing members of delicate, scrofulous and consumptive families, however, continued for some winters during that age when the body is attaining its full growth, and when cartarrhal affections are attended with the greatest danger, it may have great influence in checking the tendency to hereditary disease. Even when tubercles already exist in the lungs in a state of irritation, a residence for some years in a mild temperature, together with the adoption of a proper regimen, may be the means of allaying the irritation, and consequently of preventing the suppuration of these tubercles. By a little future attention in guarding against the known exciting causes of inflammation, these may long, and perhaps for life, remain in a state of quiescence. By such measures, and a strict adherence to the other means most proper for strengthening the constitution, and by acquiring habits calculated to inure the body to the cold and inequalities of its native climate, (among which I consider the habitual use of the cold bath as pre-eminent) I have no doubt that many lives might be saved. When, however, suppuration has actually taken place in the substance of the tubercles, my opinion is, that little or no benefit is to be expected from a change of climate in the cure of the disease; and further, that by the great and numerous inconveniencies and discomforts of so long a journey, the fatal termination of it is more frequently accelerated than protracted. That this is very frequently the case in the very advanced stages of the disease, such as I have frequently met with on the continent shortly after their arrival from England, I have no manner of doubt." P. 114.

The places whose fitness for winter residence for the consumptive are investigated by Dr. Clark, are Marseilles, Hieres, Nice, Villa Franca, Pisa, and Rome; and the plan he has followed in his examination of these appears to us very judicious. It is thus briefly stated by himself, and he seems to have faithfully adhered to it.

"I shall give in the ensuing remarks, first, a short topographical account of each, (confining myself, however, strictly to those circumstances which appear to me immediately connected with its locality in a medical point of view); secondly, observations on its climate; and, thirdly, remarks on the diseases in which it seems useful or injurious, founded on these observations, and on a knowledge of the ailments to which the inhabitants are most liable. To these will be occasionally added a few remarks on the present state of medical practice, as far as sufficient information could be gained on that point." P. 7.

Of all the places described, Marseilles seems to be, on every account, the most improper as a residence; and, indeed, the objections to it detailed by Dr. Clark are so great and obvious, that it would be truly most surprizing that it should still continue to be resorted to by any of our countrymen, were it not for the very remarkable ignorance of the profession in this country, of the nature of the climate and local habitudes of the whole south of France. This ignorance we do not say that Dr. Clark's little work will entirely remove; indeed, it is much too brief to have this effect; but it is certainly more calculated to do so than any other work we have yet met with.

Marseilles is very much exposed to the violent, cold, piercing N. W. winds (termed by the natives *mistral)* which, indeed, are felt throughout the whole northern shores of the Mediterranean with remarkable severity.

" The great evil of Marseilles, as a winter residence to the consumptive, or those liable to inflammatory affections of the chest, is the frequency and force of the dry cold northerly winds, to which, during that season, it is particularly subject, aud to the full influence of which its northerly aspect exposes it in a peculiar manner. The effect of these winds, the most frequent and violent of which is the north-west, termed by the inhabitants the *Mistral*, is to produce a very great, and often very sudden alteration of temperature. This is severely felt by the inhabitants themselves, (the surface being probably relaxed, and rendered particularly sensible, by the preceding heats,) and on many occasions cannot be guarded against by the most cautious invalid. The influence of this wind in sinking the thermometer is by no means in proportion to its effects on the living body."

This unfavourable exposure, and cold variable climate, appears to be extremely productive of disease of the pulmonary organs. Consumption is more prevalent in Marseilles than in most of the towns of France; its progress is stated to be extremely rapid; and is considered by the medical men as invariably fatal.

Females from 14 to 18 years of age are stated to be its most frequent victims. Its ravages among the youth are stated in very energetic terms by Dr. Segand, in a memoir quoted by our author. " Elle fait des ravages inouis en moissonnant la plus belle jeunesse!" P. 14.

Another evil of this place, considered as a residence for invalids, is said to be the local configuration of its vicinity, which is extremely confined and hemmed in by the neighbouring mountains, and by the thick high enclosures of the fields and gardens. P. 10.

*Hieres*, a place of much higher repute as a station for the consumptive, is admitted by our author as justly entitled to this comparative praise; but he is by no means disposed to fall in with the high opinion entertained by many of its actually great advantages in this disease. " Its exposure is better, and it is in some degree protected by its finely clothed hills from the northerly winds, which, if not warded off, are at least broken in their violence." p. 25. This is comparatively good report, but even this is weakened by a remark in the preceding page.

" It is not well sheltered from the north-east, and still less so from the north-west, which last we have noticed as blowing during the greater part of December, so as to render it unsafe for a consumptive patient to venture out of doors, except in some corner, where he is exposed to the sun's rays, and completely sheltered from it." p. 24.

Like most of the towns on the southern shores of France, Hieres is surrounded on the land side by hills, and as these lie to the north of it, they tend to shelter it much more than Marseilles. The country around it is described as being extremely beautiful and fertile, but it seems the state of the cultivation, and inconveniences of travelling, as at Marseilles, prevent invalids enjoying the advantages that the natural configuration of the place seems to afford in some situations.

" It is true, that about the bases of the hills there are some spots sheltered from the blasts of the mistral, where the invalid might enjoy several hours in the open air almost every dry day, but the difficulty is to reach them at the time that they would be most useful. The chilly blast, sweeping round every exposed corner, forbids the valetudinarian venturing there, except in a close carriage, while, on the other hand, the roads leading to these places do not admit wheeled carriages." P. 21.

Consumption is said to be much less frequent than at Marseilles.

The next place described by Dr. Clark, is *Nice*, which is also situated close on the shore, and is bounded on the whole of the land sides by an amphitheatre of lofty mountains. The topographical advantages of this place, considered as a residence for the consumptive, seem to be very considerable, and much superior to any of the stations in the south of France. From the arrangement of its mountains, above noticed,

Nice

"Nice derives the superior mildness of its climate. While open to the genial influence of the south, its mountain barrier on all other sides defends it in a great measure from the northerly winds, and particularly from the north-west or mistral. It is not altogether so well sheltered from the north, or rather north-east wind, which, sweeping down the valley of the Paglion, is not unfrequently felt during the winter and spring months with considerable severity, but in a degree much inferior, both in frequency and force, to that of the mistral in Provence." P. 29.

This superiority of the climate of Nice is shown in the luxuriance of its vegetable productions, and the extreme fertility of its soil. As a *winter* climate, indeed, Nice seems to possess every quality of equability and mildness, which are usually esteemed the most essential in the disease to which these observations refer. We fear, however, it will be found that something more is wanting to constitute Nice, or any other place, an advantageous residence for the consumptive, than the degree of mildness and equability which it seems to possess. At least from our author's account of its diseases, we are led to this conclusion. As a *Spring* climate, it seems to fail much more in these requisites.

"Nice is protected from the dreaded mistral, but is still subject to some winds, chiefly the north-east, east, and south-east, which, though they do not blow with the violence of those of Provence, nevertheless are sharp and cold. They are particularly frequent during the spring months, and form a strong objection, in my opinion, to the climate as a spring residence for the consumptive." P. 36.

Consumption is stated to be a very frequent and fatal disease at Nice; and catarrhal affections are mentioned by the medical men of the place as being their most prevalent complaints. An extract is given from an unpublished work of the celebrated Professor Foderé of Strasbourg, who spent upwards of six years at Nice, from which it appears, that this physician considers Nice as *particularly hostile* to the pulmonary organs. He considers it as beyond doubt, not only that consumption is more prevalent at Nice, and, indeed along the whole of these shores, than in most other situations, but that it is much more rapid in its progress, and fatal in its tendency.

"It seems irrefragably proved by the experience of our time, that the climate of the shores of the Mediterranean is hurtful to such invalids. I had seen a great number of these cases terminate fatally at Marseilles, and, at that time, I considered the very dry and keen winds of that place, as the principal source of evil; but I afterwards found that the warmer, softer, and more humid atmosphere of Nice, was in no respect more favourable to such complaints. Every case of hereditary tubercular phthisis proves fatal, as well at Nice as at Villa Franca, in very early age. In these places consumption is not, as in Switzerland, on the Banks of the Soane, and in Alsace, a chronic disease: on the contrary, I have very often seen it terminate in forty days. A physician of the countries just mentioned, would be surprised at the quickness with which one attack of hæmoptysis succeeds another, how readily the tubercles suppurate, and how speedily the lungs are destroyed. The English annually make fresh experience of this melancholy fact, and their burying ground in the *Croix de Marbre* too well testifies its truth." 48, 49.

Another document, quoted by Dr. Clark, of great value and interest, as illustrating and confirming his opinions of the bad effects of the climate of the northern shores of the Mediterranean, is a Thesis lately published at Edinburgh, by Dr. Sinclair, a navy surgeon, who had served several years in our fleet, in the Mediterranean, and particularly on the coasts of France. This gentleman states consumption (or at least inflammatory affections of the lungs, terminating in suppuration, and ending fatally) to be much more prevalent among our seamen on that station than elsewhere; a difference which he attributes to the extreme variability of the climate. It appears from official documents, quoted by Dr. Sinclair, that,

"During the years 1810, 1811, and 1812, there were admitted into the naval hospitals of the Mediterranean, from the fleet, (which contained about thirty thousand men,) four hundred and fifty-five cases of phthisis pulmonalis, and one hundred and forty of pneumonia,—making a proportion of one in sixty-five affected with the former complaint, or of one in fifty, when the two diseases are taken together." 52.

Upon the whole, Dr. C. seems to have left Nice with a very unfavourable impression of it as a residence for the consumptive.

*Villa Franca*, situated near Nice, only on the other side of Montalbano, is said to be still milder in point of temperature than Nice. It seems, however, to possess no essential advantages over it.

"This place, I am of opinion, must share the fate of Nice, as to its climate, whatever that may be; for though more sheltered from northerly winds, it has all the other good and bad qualities of the latter place, with the additional disadvantage of possessing few or no accommodations for invalids." 63.

*Pisa* is described as being less sheltered by mountain barriers than Nice, as having a more variable climate, and as being considerably colder; the only advantage it is stated to possess over this place is, "that there are good roads leading from it to all parts of Italy, and the invalid may leave it with safety much sooner than he could Nice."

This reminds us of the well known bon-mot of Johnson, who, when asked of the finest prospect he had seen in Scotland, mentions the *road to London!* It was, therefore, with some surprise, we found our author, in a subsequent part of his book, (p. 113) giving Pisa a decided superiority over Nice as a winter's residence for the consumptive. He certainly does not acquaint us with the reasons for this preference. The last place described from personal observation, as a station for consumptive patients, is *Rome.* The following extract will give our readers some idea of the climatorial habitudes of this famous city, as a winter residence for the consumptive.

"The climate of Rome differs considerably from that of Nice and the southern parts of Provence. It is more moist. The dry cold winds experienced at these places are comparatively little felt at Rome." 70.

"It is chiefly during the spring months, it appears to me, that Rome has the advantage in point of climate, being less liable to the keen cutting winds of the places I have already noticed. This is, however, an important circumstance, as the great difficulty is to find a good spring climate for the consumptive patient; the cold winds, which that season is liable to, I believe over the whole of Europe, being the evil most to be dreaded. At Naples they are particularly complained of. In truth, I believe no place is exempt from them; nor must I be understood as stating Rome to be so: all I wish to be understood is, that Rome is less liable to these winds, than most places I have seen." 71.

As corroborating the opinion the author had been led to form of the favourable nature of the climate of Rome, he quotes, from his own experience, several cases which appear to have been benefited by a residence there; but they are by far too few to permit us to rely much on the evidence they afford. Consumption appears to be a very prevalent disease at Rome; also inflammation of the chest and lungs. Dr. Clark does not appear to have visited *Naples,* but he has been led to consider it as much less favourable for consumptive patients than Rome. He compares it to Nice, in respect of its climate.

After these details respecting the comparative advantages and disadvantages of the various winter residences recommended for the consumptive, Dr. Clark proceeds to examine the question of a *summer residence.* He considers the whole of the south of France and Italy as improper residences during the summer months; and our experience of this disease in the warmer climates of the Globe, strongly corroborates this opinion. The following extracts from this part of the work are interesting:

"I believe I shall not err, if I say that the whole of the South of France and Italy are improper for consumptive patients during the summer months, and that the more certainly, the farther the disease is advanced: where hectic fever is formed, and ulceration of the lungs has taken place, a high temperature certainly accelerates the progress of the disease." 87.

"Though I do not think the observations of Dr. Sinclair are quite conclusive against the good effects of the climate of the Mediterranean and its shores on the generality of cases of pulmonary consumption which occur in England, and are sent abroad to pass the winter months only, they certainly show the impropriety of such patients remaining in that climate during the heats of summer, an observation which I have found corroborated by all the medical men of my acquaintance who have had opportunity of judging.

"It is rather a singular circumstance, that while the English practitioners were sending their consumptive patients to the shores of the Mediterranean, our naval medical officers navigating that sea were sending theirs to England.

"The same injurious effects in hurrying on the fatal termination of consumption, after ulceration had commenced, have been communicated to me by many of my naval friends who had served in the West Indies, as uniformly observed there. And I know that a practice, formerly prevalent in the navy, of sending seamen affected with this disease to that country, in the hope of attaining an alleviation of their complaints, has been of late years abandoned, owing to the representation of the naval medical officers of that country. From these facts, and also from my own observations in the countries themselves, I can have no doubt, nor, indeed, do I believe, is there any doubt, of the impropriety of consumptive patients remaining in the South of France and Italy during the summer months." 88.

This experience of the evil influence of the high summer heats of Italy is practically acted on by invalids, who are in the habit, it seems, of passing into Switzerland during this season, and returning to Italy in the winter. The places chiefly frequented by them are the neighbourhood of Lausanne and Geneva, which appear to possess no other claim to preference, than the circumstance of being cooler than Italy, and in lying conveniently in the route to that country. The invalids are recommended to reach Switzerland in the middle of June, and to leave it in the end of September. In the concluding portion of Part I. Dr. C. protests loudly against the practice of sending patients, in a late stage of consumption, abroad. He details several striking and melancholy instances of this cruel measure from the stores of his own experience. The following extract does equal honour to his understanding and his heart; we earnestly call the attention of our readers to it.

"During my residence on the continent, I have had frequent occasions to remark with surprize the very advanced stages of the disease in which many of our consumptive patients were sent abroad. This is the more remarkable, as, however medical men may differ about the propriety of sending such patients abroad in the earlier, there surely ought to be no question about its impropriety in the latter stages. For my own part, I have seen enough to convince me that it is not only a very useless, but often a very cruel thing, to banish such patients from all the comforts of home, and send them forth to undertake a long journey through a foreign country, deprived probably of all they hold dearest to them, and without those thousand nameless comforts by which the watchful care of friends may cheer even the last period of a hopeless disease. The medical man who reflects on the distresses that such patients must be liable to during such a journey, arrested perhaps in their progress by the increase of some of those symptoms which attend the advanced stages of consumption, in very indifferent accommodations probably, and far from any medical advice in which they can confide, will surely long hesitate ere he condemns the fated victim of this remorseless malady to the additional evils of expatriation: and his motives for hesitation will be increased, when he considers how often the unfortunate patient sinks a prey to his disease, long before he reaches the place of his destination; or, at best, arrives at it in a much worse condition than when he left England, and doomed, shortly, to add another name to the long and melancholy list of his countrymen that have sought out, with pains and suffering, a distant country, only to gain in it an untimely grave!" 117.

In dwelling on the highly important subject of the effects of foreign climates on consumption, we have carried our extracts to such a length as to prevent our noticing many other interesting matters in Part First, especially a capital Memoir on *Cretinism* and *Goitre*, and a Report on the *Malaria Fevers of Rome*. The same circumstance makes us content ourselves with a few extracts only from Part Second. This part consists of Notes on several of the Parisian Hospitals, and on the Hospitals and Medical Schools of Lyons, Strasbourg, Turin, Genoa, Pavia, Padua, and Bologna. Of the hospitals of Paris he notices La Charité, St. Louis, Maison des Enfans Malades, Hôpital Necker, Val-de-Grace, Salpetriere et de Clinique Interne. In his notes on La Charité, he confines his remarks chiefly to the practice of Dr. Esquirol, in giving the nux vomica in paralysis. This seems to be a powerful but very dangerous remedy. Several patients have been killed by its use. The following extract from the account of the Maison des Enfans is interesting, and we may add, is very flattering (by way of contrast) to the practice in this country.

"In the spring of 1818, small-pox was very frequent in Paris, and very fatal; more than half of those admitted into this hospital sometimes dying.

"Strange to say, the heating and sudorific plan was the one adopted at this time in the treatment of these little patients! All those who died were examined, and the appearances observed were inflammation and ulceration of the internal coat of the intestines, pustular eruptions there, or on the surface of the peritoneum; and, in three cases, a false membrane, similar to that of croup, was found lining the whole alimentary canal from the œsophagus to the rectum: in these three cases, the eruption on the surface had never shown itself distinctly. These morbid appearances called the attention of the medical officers to Dr. Broussais' opinions; the cooling antiphlogistic treatment, with the application of leeches over the abdomen, was adopted in consequence, and the speedy reduction of the mortality soon evinced the superiority of the practice.

"The following statement of the proportion of deaths from this disease is interesting on many accounts; and taken in conjunction with the circumstance just detailed, cannot fail to make a strong impression on the mind of the English Reader.

"*Small-pox Cases admitted into the Hospital des Enfans Malades, in* 1816, 1817, *and* 1818.

| | Cured. | Died. | Total. |
|---|---|---|---|
| 1816 - - - - - - - - - - - - - - | 42 - - - - - - | 53 - - - - | 95 |
| 1817 [From Jan. to Aug.] | 51 - - - - - - | 37 - - - - | 88 |
| 1818 - - - - - - - - - - - - - - | 117 - - - - - - | 102 - - - | 219 |
| | 210 | 192 | 402" |

Page 150.

In his account of L'Hopital Necker he dwells chiefly on the discovery of Dr. Laennec, on which we shall say nothing, having said so much in our last number. We are happy to see our opinion of the utility of Dr. L's diagnostics corroborated by that of so intelligent a practitioner as Dr. Clark.

In his notes on the Val-de-Grace, he gives an account of the doctrines and practice of Dr. Broussais, an exposition of which we expect soon to lay before our readers in detail. We cannot help remarking, however, at present, that, like most theorists, this eminent physician is too exclusive and confined in his mode of explaining morbid action.

In almost every disease that affects the human body,

Dr. B. can see nothing but inflammation of the mucous membrane of the stomach and intestines.

Dr. Clark appears to entertain but an indifferent opinion of the *practice* followed in the Parisian hospitals.

" The practice appeared to me particularly inert: ptisanes pectorales, eau gommé, lavement emollient, &c. were among the most active prescriptions, and few escaped without some one or two of these. 'Tis sickening to an English physician, in visiting the hospitals of Paris, to hear nothing but these eternal ptisans ordered for every patient, let the disease be chronic or acute. It may be said they are harmless at any rate, and may satisfy the patient that something is doing. But surely the latter consideration is little necessary in hospital practice, and I am inclined to think that the plan is often worse than harmless, as it operates, in some degree, on the mind of the physician, and leaves an impression that he has done something, when, in truth, he has done nothing. In this way, perhaps, it prevents the employment of more active means when they are actually requisite." 170.

He seems to us to account pretty well for this state of things.

" Generally speaking, the physicians in the hospitals of Paris (as appears to me) have too many patients to attend. Though it was summer when I last visited them, and there were extremely few fevers and acute diseases, yet Dr. Fouquier, of La Charité, had one hundred and four patients under his own charge, with one assistant only. Dr. Recamier, of the Hotel Dieu, had one hundred and ten, and the physicians of St. Louis had a still larger proportion.

" The fatal cases are generally examined after death. Dr. Fouquier told me, that for twelve years that he had been physician to La Charité, no patient had died without being examined.

" It is allowed that post-mortem examinations, when properly conducted, form one of the principal means of enabling the physician to acquire an intimate knowledge of the nature of disease, and to lead to those principles which are to guide him in the treatment of it. But the knowledge derived from these examinations, with whatever care they may be performed, when taken by itself, is but of little real utility. If the previous history of the disease be not accurately known, little is to be expected from them; and it is a deficiency of this knowledge that appears to me the principal circumstance which has rendered these examinations at the hospitals of Paris of so little real value. I have attended not a few of these examinations, and seen the surprize excited at some appearances, which, had the morbid phenomena during the course of the disease been carefully observed, must have been known at least, if they could not have been remedied; and I have also had occasion to remark the difference that sometimes occurred between the physician and assistants with respect to previous symptoms. All this convinced me how truly useful these researches might have proved but for the want of information relative to the history and progress of the disease. This circumstance, however, certainly cannot be sufficiently attended to, where the physician has so many patients under his care." 130, 131.

An interesting and full account is given of the hospitals at Lyons. It appears that the antiphlogistic mode of treating febrile disease, is making gradual progress here also, and not before it is time, since Dr. C. informs us, that *even now*, four-fifths of the practitioners in this city are Brunonists! We quote the following statement in justice to our continental brethren.

"In this gentleman (Dr. Desgaultières) I found a very enlightened physician, who had had much experience, both in the army and in private and hospital practice; and, in the present unsettled state of the opinions of medical men about the cause of fever, his may be stated here with propriety. I do this the more readily, as it appeared to me that his opinion was formed not from any theoretical notions, but from careful and extensive observation, and because it approximates so closely to that which the great increase of pathological researches has led so many British practitioners of late to adopt. His idea is, that fever always depends upon local affection, irritation or inflammation of some organ, or even an unusual accumulation of blood (congestion) in some viscus, without absolute inflammation. He is far from limiting such local affection to the gastro-enteritic system with Dr. Broussais; yet his conviction is, that fever invariably depends upon, and has its origin in some local affection, and that from this we ought to set out in our explanation of its phenomena." 183.

Dr. Clark's account of the Medical School of Strasbourg is extremely meagre. Its prosperity seems on the decline; "a few years ago, the number of medical students amounted to from four to five hundred; at present, there are only about two hundred, and from thirty to forty only take their degrees annually." The Italian schools seem somewhat more flourishing. Among these, Bologna ranks as one of the first.

"Medicine at Bologna is cultivated with assiduity, and the Professors are men of established reputation. Tommassini, Professor of Medicine, is well known as the author of an excellent work on Yellow Fever, and several others of less note. He is one of the great supporters of the new Italian, or contra-stimulant doctrine, as it is called, and which, in practice at least, is directly opposed to the system of Brown, which for a long time was advocated warmly in Italy. The latter, it is well known, considered almost all diseases as arising from debility, and requiring stimulants, while the present doctrine, 'Nuova Dottrina Medica Italiana' looks upon most diseases as arising from increased action, and requiring depriments—contra-stimulants. The Medical School of Bologna may be considered at the head of this new doctrine, and the Professors its most able and zealous advocates." 193.

Dr. C. gives a full detail of the plan of medical study prescribed by the University, which seems very judicious. The course extends to four years.

" The mode of examination of candidates for the different degrees seems very judicious, and is considerably different from that followed in our medical schools. Five professors constitute a quorum; and at the examination of a candidate, each submits to him twenty different subjects, taken from his own particular course of instruction. He draws one of these by lot, and is forthwith examined upon it, whatever it may be. At the termination of the examination, the professors give their votes secretly, and the number of these obtained, and the mode in which they are obtained by the candidates, constitute three different modifications of the same degree, which are expressed in the diplomas.

" If they obtain two-thirds of the votes they are declared *approved;* if the whole, they are declared *unanimously approved* (a pieni voti); and to express a still further praise, they are declared *honourably approved*—(approvati conlodi). Those who are rejected cannot present themselves till after another year's study.

" I was told that the plan of study just described is about to be changed, by an order from Rome." 199.

" There are altogether about two hundred medical students at the University of Bologna; but of these, a small proportion only aspire to the higher ranks of the profession. Many, I found, were merely preparing themselves for being apothecaries, and many to practise the lower branches of surgery—' Bassa Chirurgia,'—a class in the profession unknown among us, and holding a rank something similar to the ' officiers de santé,' in France.

" This system of sending forth a set of half educated men, who, no doubt, as in other countries, overstep in their practice, the limits prescribed to them, is, with justice, complained of by the regularly educated members of the profession in Italy. The Germans are blamed for introducing this practice. A system more calculated, indeed, to bring the profession into disrepute, cannot well be imagined; and it may be one, among several other causes, of the very different rank in society which the medical men of Italy hold, compared with those in England. But a still stronger reason may be found, I apprehend, in the system of gratuitous medical education in use in the former country. The students attend the lectures and hospitals almost for nothing, and the graduation fees are very trifling. The annual salary of the medical professors themselves, amounts only to about £. 150. The facility thus afforded to medical students of acquiring their profession on easier terms than the lowest mechanic can his trade, so common on the continent, and of which they boast, though it may have some advantages, is, upon the whole, I am inclined to believe, injurious to the profession." 196.

" *Padua.* The University of Padua at present holds a deservedly high character in Italy, as a medical school. Within the last few years, the number of students has considerably increased, and the present building being found too small, the Emperor, on his late visit to Italy, gave orders for the erection of a new one, on a more extensive scale. The plan of medical studies in the University of Padua is good. It extends to five years; and two courses of lectures are delivered in each Session." 204.

The following extract respecting Pavia will be interesting to all who venerate (and who does not venerate) the name of the great man, with whose name that of his city must be eternally associated in the memory of the votary of medicine.

" Every thing about the Medical School of Pavia is very perfect, and the musea, class rooms, &c. all arranged and fitted up in a very superior style; yet I was surprized to find every thing on so small a scale at a University, which, at one time collected so great a concourse of students from all parts of Europe, and boasted of some of the first medical professors in the world.

" As a medical school, Pavia is now much fallen in estimation. The number of medical students of all classes in the session of 1817 and 1818, I was told, amounted to nearly four hundred; and that about forty annually took the higher degrees of the profession. The wards of the general and clinical hospital were in good order, but small. We must not, however, forget that the town is but small, containing only about 20,000 inhabitants. This is a circumstance which must always have been against Pavia as a school of practical surgery. It ought, however, to add not a little to the merit of the venerable Scarpa, whose works have added so largely to the stock of surgical knowledge, and to whom this University is so much indebted.

" Scarpa is now the Director of the Medical Department of the University; the plan of medical study at which, I shall not mention, as he informed me they are about to change it. Scarpa's sight has failed him much, yet he appears to enjoy good health, and a flow of spirits and cheerfulness not often met with at his age. Although in a great measure retired from the exercise of his profession, he has lost nothing of his enthusiasm for it. In his library, I found all the best English medical works. He complained much of the very tedious manner in which he received publications from our country, being obliged frequently to get them round by Germany.

" Whoever visits this great man will, in addition to the pleasure and information derived from his animated conversation, find in his house a collection of some of the finest paintings by the first Italian masters; for Scarpa is an admirer and an encourager of the fine, as well as the useful arts." 210.

The whole of the establishments in the Sardinian dominions seem to wither beneath the deadly sway of an ignorant and despotic government. We shall conclude this article by the following most horrible account of the lunatic asylum (Casa de' Pazzi) of Turin.

"My expectations of this Establishment were not raised very high, from the state in which I found that which I have just mentioned, and still less from some conversation I had with one of the medical professors of the University the evening preceding my visit, (during which I perceived evidently a wish that I should not see it,) but I never could have anticipated the dreadful spectacle of tortured humanity which was here exhibited, and of which any description I can give must come far short of the impression it made upon my mind. I shall simply state the situation of the wards and patients as I found them at the time of my visit.

"The part of the hospital we were first taken to, consisted of small rooms, similar to those generally met with in such institutions, but I was disappointed to find these were not for the poor patients, but for those who paid a certain sum for being kept. The first of these that was opened presented to us the wretched prisoner perfectly naked, and chained down to his bed by both wrists. He had raised himself up in his bed, as far as his chains admitted, by which movement he had thrown off the single coverlet that had been cast over him. He had no shirt; his legs (apparently red and swollen from cold) were drawn up under the corner of the bed cover, which lay over a small part of his body; he was pale and emaciated; he uttered not a word. In short, a human being in so wretched a state I had never before seen; but I was soon to witness others in a state still more horrible.

"We were next conducted into a ward where thirty beds were huddled together, on most of which lay a poor wretch chained by one or more limbs to the bedstead; for to each corner of these was attached a massy iron chain, with a clasp of the same materials and strength at the extremity, for admitting the wrists and ancle; and, according as the keeper judged necessary, one or more of these were applied. Some were polished, and bright as silver, from constant use. I imagined these were their most unruly patients, but was told that this was by no means the case.

"To these we were next led; and on the unbolting of the door of a large cell, the scene that presented itself almost exceeds belief. The spectacle of the poor wretches, naked, or covered only by some straw, chained down hand and foot to their bedsteads, the clanking of their chains, the dreadful vociferations they set up at the sight of him who rivetted these chains, and still more, the horror excited by such a spectacle, no terms are strong enough to depict! I had read and heard of chains and other means of torture for subduing (irritating,) the unfortunate maniac; I had even seen such singly chained to the wall by the neck, like an infuriated and dangerous beast; but a den like this, crowded and crammed with human beings, chained down, without a rag of covering, struggling to raise their heads, and exhibiting their emaciated and galled limbs from the heap of straw that had been thrown upon them, was a scene I never expected to witness, and which I hope in God I may never witness again! After visiting similar establishments in Paris, and some in our own country, where chains have been laid aside, and a mild system of treatment adopted, with a success which (setting humanity out of the question) more than warrants its continuance, I was ill prepared to meet such a dreadful contrast on crossing the Alps. In this cell there were twelve men, three of whom only were allowed any thing more than straw to cover them. Some I was told had been confined there for many months. On approaching them, they exhibited their chained limbs with the most earnest entreaties for liberation. One man had two chains on one arm. In this case, the space between the iron clasps was red, swollen, and ulcerated, and the mortification, which, in all probability was to follow, would soon render chains unnecessary for him. Others had their limbs galled, but not in such a degree as that described. In one instance only, in the whole hospital, did I observe any thing introduced between the iron ring and the limb. The rest of the mens' wards were similar to that I first noticed.

" From the mens' we were led into the womens' department, which was in the higher part of the house, and which, in every respect, we found similar to that we had just left, the beds huddled closely together, chains always ready, many applied, and most of the beds occupied; for whether to save trouble, or from the poor creatures having no clothes but the coverlet that was thrown over them, almost every one was in bed. Here, as below, was also a cell, where straw afforded the only covering, where the chains were more heavily applied, and where the state of furious desperation to which the wretched victims were driven, was expressed in terms equally violent and still more affecting. One of these tortured women, held up her arm which was raw and had been bleeding, from the iron clasp having worked its way into the flesh!

" Such is the dreadful state of this house, which contains one hundred and eighty males and ninety-seven females; of whom one-third, the keeper told me, were kept *constantly* chained. From the same source I learned, that the annual number of deaths (and this I apprehend is the principal way in which this house gets rid of its inhabitants;) sometimes amounted to eighty (nearly one-third of the whole); that they *had* been as few as thirty; and that the average was fifty, (nearly one-fifth—a mortality I believe unequalled in any institution of the kind in any country.

" With respect to the murderous receptacle for the deranged, I know not what relief to propose; nothing but a perfect change, I fear, can remedy the state it is in; perhaps it would even be for the benefit of humanity, that the same dreadful remedy were applied to it as was formerly applied to the Hotel Dieu of Paris; and could it rise from its ashes in *Piedmont*, as that hospital did in France, he who applied the torch would deserve to be ranked among the benefactors of his country and mankind. In its present state, it is an infamous disgrace to the Government under which it exists: I say Government, because, in a despotic one, such as that of Piedmont, where private opinion dares not be expressed, the Government alone is responsible for the state of all such institutions. But these things are beneath the notice of a King of Sardinia (who, by the way, is particularly bound to visit La Carita in person; but he prefers a deputy, who again, I fear, employs a substitute, in imitation of his magnanimous sovereign). The royal family of France are not ashamed, in their visits to the departments, to look into the hospitals, and even to administer consolation at the bed-side of their subjects; and the Emperor of Austria, in his late journeys, visited the hospitals, as well as other public institutions: but such is the horror of an hospital at the court of Sardinia, that a physician who has entered one, is not suffered to cross the precincts of the palace, lest he should carry the seeds of infection among the royal family!" 223.

After this ample exhibition of interesting specimens of Dr. Clark's volume, in presenting which to our readers, we have endeavoured to minister to their gratification, while we offered a fair view of the work itself, it is hardly necessary for us formally to express our opinion on its merits. These certainly are very considerable, but they are far from being without alloy. The greatest fault of the work is its brevity. Of some very interesting scenes, so very little is said, that we cannot easily acquit the author of negligence, or want of curiosity, during his visits to them. He, indeed, tells us in his preface, that he professes to give us only "detached notes;" and seems to consider, that we ought to be thankful for what he gives us. We ARE thankful; yet, we repeat, that we would have been more satisfied had he given us *no* information respecting some of the places, than we are by the meagre morsels which he has vouchsafed to give us from the rich and overflowing granaries to whose half-opened portals he conducts us. We trust, however, that before Dr. C. finally revisits his native country,* he will be enabled to fulfill the hopes expressed in the Preface, of filling up the outline which he has chalked out in the present Essay.

"At all events, (he says) this Essay will be useful to myself, as a sort of text book for future observations of a like nature, and as a basis whereon, I hope, more extensive opportunities may enable me to rear a superstructure more creditable to myself, more commensurate with the importance of the subject, and more worthy the attention of the public."

---

* Dr. Clark, we understand, is at present residing at Rome, of which city he is preparing a Medical Topography, which cannot fail to be highly interesting.

